# Prevalence of diabetes and hypertension and their interaction effects on cardio-cerebrovascular diseases: a cross-sectional study

**DOI:** 10.1186/s12889-021-11122-y

**Published:** 2021-06-25

**Authors:** Zhehui Wang, Tubao Yang, Hanlin Fu

**Affiliations:** 1 Department of Epidemiology and Health Statistics, XiangYa School of Public Health, Central South University, Changsha, Hunan Province China; 2 Hunan Provincial Key Laboratory of Clinical Epidemiology, Changsha, China; 3 Department of Obstetrics and Gynecology, The First Affiliated Hospital of Zhengzhou University, Zhengzhou, 450052 China

**Keywords:** Diabetes, Hypertension, Stroke, Coronary heart disease, Interaction

## Abstract

**Background:**

Hypertension and diabetes mellitus are two of the major risk factors for cardio-cerebrovascular diseases (CVDs). Although prior studies have confirmed that the coexistence of the two can markedly increase the risk of CVDs, few studies investigated whether potential interaction effects of hypertension and diabetes can result in greater cardio-cerebrovascular damage. We aimed to investigate the prevalence of hypertension and diabetes and whether they both affect synergistically the risk of CVDs.

**Methods:**

A cross-sectional study was conducted by using a multistage stratified random sampling among communities in Changsha City, Hunan Province. Study participants aged > = 18 years were asked to complete questionnaires and physical examinations. Multivariate logistic regression models were performed to evaluate the association of diabetes, hypertension, and their multiplicative interaction with CVDs with adjustment for potential confounders. We also evaluated additive interaction with the relative excess risk ratio (RERI), attribution percentage (AP), synergy index (SI).

**Results:**

A total of 14,422 participants aged 18–98 years were collected (men = 5827, 40.7%). The prevalence was 22.7% for hypertension, 7.0% for diabetes, and 3.8% for diabetes with hypertension complication, respectively. Older age, women, higher educational level, unmarried status, obesity (central obesity) were associated with increased risk of hypertension and diabetes. We did not find significant multiplicative interaction of diabetes and hypertension on CVDs, but observed a synergistic additive interaction on coronary heart disease (SI, 1.43; 95% CI, 1.03–1.97; RERI, 1.94; 95% CI, 0.05–3.83; AP, 0.26; 95% CI, 0.06–0.46).

**Conclusions:**

Diabetes and hypertension were found to be associated with a significantly increased risk of CVDs and a significant synergistic additive interaction of diabetes and hypertension on coronary heart disease was observed. Participants who were old, women, highly educated, unmarried, obese (central obese) had increased risk of diabetes and hypertension.

**Supplementary Information:**

The online version contains supplementary material available at 10.1186/s12889-021-11122-y.

## Background

Hypertension (HT) and Diabetes mellitus (DM) have been confirmed as two of the major risk factors for cardio-cerebrovascular diseases (CVDs) [1, 2]. It has been found that individuals with both DM and HT have a greater risk of cardio-cerebrovascular disease than those with only one condition [3]. However, few studies investigated the interaction of DM and HT on the risk of CVDs [4]. It is not yet clear whether the increased risk resulting from the coexistence of DM and HT could be attributed to a simple combination or their interaction.

DM and HT share common comorbidities [5–7]. Their frequent coexistence is not a coincidence but due to some shared pathogenic mechanism. Diabetic patients are twice as likely to have HT as non-diabetic patients. Similarly, patients with HT are more likely to develop diabetes than those with normotensive people. In China, nearly 100 million people have HT, nearly 30 million people have DM, and approximately 15 million people have both HT and DM [8].. The major outcome of this comorbidity is cardio-cerebrovascular diseases, which account for nearly half of the death causes.

In general, physicians pay more attention to the associations between risk factors and outcomes. Thus, the interaction is often neglected. The term “interaction” refers to the fact that at different levels of a risk factor, another risk factor has different effects on the disease, and vice versa [9]. Additive interaction scale and multiplicative interaction scale are usually used to measure the interactions between factors. Additive interaction refers to the effect of two (or more) factors acting on a disease is greater (synergism) or less (antagonism) than the sum of the independent effects of these factors. Multiplicative interaction refers to the effect of two (or more) factors acting on a disease is greater (positive) or less (negative) than the product of the independent effects of these factors.

Given the high prevalence of DM and HT and their strong association with CVDs, our study aimed to determine whether HT and DM act synergistically towards cardio-cerebrovascular diseases in a general population.

## Methods

### Study participants and baseline survey

This cross-sectional study was based on National Chronic Disease Management Survey conducted in Changsha, the capital of Hunan Province, in 2012. A multistage stratified random sampling method was used to select participants from the community population. In the first stage, 5 districts were randomly selected (Liuyang, Ningxiang, Kaifu, Tianxin, Furong) from a total of 9 districts in Changsha. In the second stage, 2 subregions were selected (streets/towns) from each of the five geographical locations (East, West, South, North, and Middle) in the 5 selected districts. In the third stage, 2 communities/villages were randomly selected from each of the selected subregions. In the final stage, 150 households were randomly selected from each selected community/village and one household resident with age > = 18 was selected as the study participants. Therefore, a total of 50 subregions, 100 communities/villages, and 15,000 household/participants were selected. Of those selected, 14,422 (96.1%) participants completed the baseline survey and were included in the current analysis. The study was submitted to the Ethics Committee of Xiangya School of Public Health, Central South University and was granted a waiver beacause this study used the data from 2012 national chronic disease management project and did not involve the human trial or personal information. Informed consent was obtained from the participants before the investigation. More details of the study design were previously published [10].

The baseline in-person interview was conducted by trained interviewers (Additional file 1: Appendix 1). Smoking status was categorized as never-smoking, current smoking (at least one cigarette per day), and former smoking (quit smoking more than 12 months). Current drinkers were those who drank once or more in the recent month. Regular exercisers were those who exercised at least three times a week. The disease histories included the status of diabetes, hypertension, coronary heart disease, and stroke.

### Physical examination and disease definition

Height and weight measurement: the subjects took off their coats, shoes, and hats, stood on the automatic measuring instrument with feet 45 degrees apart, and heels close to the instrument as far as possible. The instrument automatically reads the measurement results of height and weight. BMI = weight (kg) / height (m)^2^. BMI ≧28.0 was defined as obesity according to the Chinese standard [11].

Waist circumference measurement: the subjects take off their coats, loosen their belts, stand naturally on both legs and keep calm breathing. The medical staff used a tape measure to horizontally circle the abdomen of the subjects to measure the waist circumference. Central obesity was defined as a WC > 90 cm for men and > 85 cm for women [12].

Blood pressure measurement: the subjects sit in the room for 5 min, keep calm and then start to measure. The medical staff used an automatic blood pressure meter to measure. During the measurement, the subjects kept the arm position at heart level, and the instrument automatically read the blood pressure value. According to the Chinese hypertension guidelines, hypertension was defined as systolic BP (SBP) ≥ 140 mmHg, diastolic BP (DBP) ≥90 mmHg, or a history of diagnosed hypertension [13].

Blood glucose measurement: After fasting for 12 h, a trained blood collector took fasting blood from each subject. The fasting blood glucose was detected by hexokinase method within 2 h of venous blood extraction. According to the American Diabetes Association [14], Diabetes was defined as blood glucose ≥7.0 mmol/L or a history of type 1 diabetes or type 2 diabetes.

The history of CVDs, including CHD and stroke, was self-reported. CHD included ischemic coronary heart disease, myocardial infarction, angina pectoris, coronary artery bypass grafting, percutaneous coronary thrombolysis, and coronary angioplasty. Stroke included ischemic stroke and hemorrhagic stroke.

### Statistical analysis

Means and standard deviations were reported for continuous variables and frequencies and percentages were reported for categorical variables. The chi-square test or t-test was used to compare the differences in demographic characteristics between patients and normal people. The status of DM and HT can be divided into four categories: a) non-DM & non-HT group: normotensive and normoglycemic participants; b) DM & non-HT group: participants with diabetes only; c) non-DM & HT group: participants with hypertension only; d) DM & HT group: participants with both diabetes and hypertension. The multiplicative interaction between HT and DM on CVDs was evaluated using logistic regression models with adjustment for potential confounders determined with Directed Acyclic Graphs (DAGs) [15]: The additive interaction between DM and HT on CVDs was evaluated with relative excess risk ratio (RERI), attribution percentage (AP), and synergy index (SI). The confidence intervals (CIs) of the above indexes were estimated [16]. *P* < 0.05 (two-sided) was considered statistically significant. Data with missing values were deleted. All statistical analyses were performed with SPSS for Windows 18.0 (SPSS Inc., Chicago, IL, USA).

## Results

### Demographics

The demographic characteristics of the participants classified by disease status were summarized in Tables 1 and 2. Of the 14,422 participants, 40.7% were male with a mean age of 53.84 years and the range of 18–98 years. The proportions of participants who were current smokers, current drinkers, having regular exercise, having obesity and central obesity were 25.8, 20.1, 33.2, 8.6, and 26.0%, respectively. The mean values of age, the proportion of females, education level of primary school and below, former smokers, non-current drinkers, regular exercisers, obese and central obese people were significantly higher in CHD patients than normal people (*P* < 0.001). Similar results were found in the other four diseases except for stroke (*P* < 0.05). We found that only the mean values of age and percentages of former smokers, non-current drinkers, regular exercisers, and central obesity were significantly higher in stroke patients than normal people (*P* < 0.05).

**Table 1:** Basic demographic characteristics of participants by cardio-cerebrovascular diseases (CVD) status (*n* = 14,422)

Variables	Total	CHD			Stroke			Total CVDs		
		Patients	Non-patients	*P*	Patients	Non-patients	*P*	Patients	Non-patients	*P*
Age (year)^*^	**53.84 ± 14.88**	64.71 ± 12.28	52.96 ± 14.73	< 0.001	64.52 ± 13.46	53.68 ± 14.85	< 0.001	64.74 ± 12.23	52.85 ± 14.71	< 0.001
**Gender,** ***n*** **(%)**										
Men	**5927 (41.1)**	381 (35.4)	5546 (41.6)	< 0.001	95 (45.5)	5832 (41.0)	0.197	444 (37.0)	5483 (41.5)	0.002
Women	**8495 (58.9)**	696 (64.6)	7799 (58.4)		114 (54.5)	8381 (59.0)		757 (63.0)	7738 (58.5)	
Educational level^*^, ***n*** **(%)**										
Primary school or below	**5909 (41.1)**	657 (61.3)	5252 (39.5)	< 0.001	93 (44.7)	5816 (41.0)	0.412	707 (59.2)	5202 (39.4)	< 0.001
Middle school	**7090 (49.3)**	337 (31.4)	6753 (50.7)		93 (44.7)	6997 (49.4)		400 (33.5)	6690 (50.7)	
College or above	**1384 (9.6)**	78 (7.3)	1306 (9.8)		22 (10.6)	1362 (9.6)		88 (7.4)	1296 (9.8)	
Marital status^*^, ***n*** **(%)**										
Unmarried	**702 (4.9)**	26 (2.4)	676 (5.1)	< 0.001	4 (1.9)	698 (4.9)	0.007	28 (2.3)	674 (5.1)	< 0.001
Married	**11,944 (83.1)**	807 (75.2)	11,137 (83.7)		167 (80.3)	11,777 (83.1)		905 (75.6)	11,039 (83.9)	
Divorced or widowed	**1730 (12.0)**	240 (22.4)	1490 (11.2)		37 (17.8)	1693 (11.9)		264 (22.1)	1466 (11.1)	
Smoking status^*^, ***n*** **(%)**										
Never	**10,054 (70.1)**	770 (71.8)	9284 (69.9)	< 0.001	148 (71.2)	9906 (70.0)	< 0.001	857 (71.6)	9197 (69.9)	< 0.001
Former	**600 (4.2)**	94 (8.8)	506 (3.8)		19 (9.1)	581 (4.1)		104 (8.7)	496 (3.8)	
Current	**3696 (25.8)**	209 (19.5)	3487 (26.3)		41 (19.7)	3655 (25.8)		236 (19.7)	3460 (26.3)	
Current drinking^*^, ***n*** **(%)**										
Yes	**2887 (20.1)**	157 (14.6)	2730 (20.5)	< 0.001	26 (12.4)	2861 (20.2)	0.006	173 (14.4)	2714 (20.6)	< 0.001
No	**11,507 (79.9)**	918 (85.4)	10,589 (79.5)		183 (87.6)	11,324 (79.8)		1026 (85.6)	10,481 (79.4)	
Regular exercise^*^, ***n*** **(%)**										
Yes	**3678 (33.2)**	380 (41.4)	3298 (32.5)	< 0.001	83 (59.3)	3595 (32.9)	< 0.001	419 (42.5)	3259 (32.3)	< 0.001
No	**7397 (66.8)**	537 (58.6)	6860 (67.5)		57 (40.7)	7340 (67.1)		568 (57.5)	6829 (67.7)	
Obesity^*^, ***n*** **(%)**										
Yes	**1242 (8.6)**	130 (12.1)	1112 (8.3)	< 0.001	17 (8.1)	1225 (8.6)	0.800	140 (11.7)	1102 (8.3)	< 0.001
No	**13,164 (91.4)**	946 (87.9)	12,218 (91.7)		192 (91.9)	12,972 (91.4)		1060 (88.3)	12,104 (91.7)	
Central obesity^*^, ***n*** **(%)**										
Yes	**3731 (26.0)**	420 (39.1)	3311 (24.9)	< 0.001	74 (35.4)	3657 (25.8)	0.002	462 (38.6)	3269 (24.8)	< 0.001
No	**10,644 (74.0)**	653 (60.9)	9991 (75.1)		135 (64.6)	10,509 (74.2)		735 (61.4)	9909 (75.2)	

^*^ The variable contained missing values.

**Table 2:** Demographic description of participants by the status of HT or DM (*n* = 14,422)

Variables	DM			HT			DM complicated with HT		
	Patients	Non-patients	*P*	Patients	Non-patients	*P*	Patients	Non-patients	*P*
**Age (year)**	60.19 ± 12.92	53.37 ± 14.91	< 0.001	61.34 ± 12.85	51.65 ± 14.72	< 0.001	61.73 ± 13.35	53.53 ± 14.85	< 0.001
**Gender,** ***n*** **(%)**									
Men	345 (34.4)	5582 (41.6)	< 0.001	1241 (38.1)	4686 (42.0)	< 0.001	178 (32.5)	5749 (41.4)	< 0.001
Women	658 (65.6)	7873 (58.4)		2017 (61.9)	6478 (58.0)		370 (67.5)	8125 (58.6)	
**Educational level,** ***n*** **(%)**									
Primary school or below	478 (47.7)	5431 (40.6)	< 0.001	1658 (51.0)	4251 (38.2)	< 0.001	254 (46.4)	5655 (40.9)	0.018
Middle school	442 (44.1)	6648 (49.7)		1332 (41.0)	5758 (51.7)		238 (43.4)	6852 (49.5)	
College or above	82 (8.2)	1302 (9.7)		261 (8.0)	1123 (10.1)		56 (10.2)	1328 (9.6)	
**Marital status,** ***n*** **(%)**									
Unmarried	37 (3.7)	665 (5.0)	0.001	102 (3.1)	600 (5.4)	< 0.001	25 (4.6)	677 (4.9)	< 0.001
Married	807 (80.8)	11,137 (83.3)		2556 (78.7)	9388 (84.4)		423 (77.5)	11,521 (83.3)	
Divorced or widowed	155 (15.5)	1575 (11.8)		590 (18.2)	1140 (10.2)		99 (17.9)	1632 (11.8)	
**Smoking status,** ***n*** **(%)**									
Never	752 (75.1)	9302 (69.7)	< 0.001	2336 (71.9)	7718 (69.5)	< 0.001	424 (77.5)	9630 (69.8)	< 0.001
Former	52 (5.2)	548 (4.1)		217 (6.7)	383 (3.5)		32 (5.9)	568 (4.1)	
Current	197 (19.7)	3499 (26.2)		697 (21.4)	2999 (27.0)		91 (16.6)	3605 (26.1)	
**Current drinking,** ***n*** **(%)**									
Yes	122 (12.2)	2765 (20.6)	< 0.001	503 (15.5)	2384 (21.4)	< 0.001	63 (11.5)	2824 (20.4)	< 0.001
No	880 (87.8)	10,627 (79.4)		2748 (84.5)	8759 (78.6)		485 (88.5)	11,022 (79.6)	
**Regular exercise,** ***n*** **(%)**									
Yes	381 (49.3)	3279 (32.0)	< 0.001	1033 (40.4)	2645 (31.0)	< 0.001	219 (53.2)	3459 (42.4)	< 0.001
No	392 (50.7)	7005 (68.0)		1523 (59.6)	5874 (69.0)		193 (46.8)	7204 (67.6)	
**Obesity,** ***n*** **(%)**									
Yes	858 (85.7)	12,306 (91.8)	< 0.001	2808 (86.3)	10,356 (92.9)	< 0.001	452 (82.8)	12,712 (91.7)	< 0.001
No	143 (14.3)	1099 (8.2)		446 (13.7)	796 (7.1)		94 (17.2)	1148 (8.3)	
**Central obesity,** ***n*** **(%)**									
Yes	569 (56.8)	10,075 (75.3)	< 0.001	1953 (60.1)	8691 (78.1)	< 0.001	274 (50.1)	10,370 (75.0)	< 0.001
No	432 (43.2)	3299 (24.7)		1296 (39.9)	2435 (21.9)		273 (49.9)	3458 (25.0)	

### Prevalence of DM and HT

Table 3 showed the prevalence of DM and HT by gender and age. The overall prevalence was 22.7% for HT, 7.0% for DM, and 3.8% for DM with HT complication, respectively. The prevalences were positively correlated with age in general, with the peak value of 66–75 years old, and then decreased. Of note, the prevalences were relatively high in the young participants aged from 18 to 25. Stratified by sex, we found that women had a higher prevalence of hypertension than men in age groups before 36–40 years old (*P* < 0.001).

**Table 3:** Prevalence (%) of DM and HT in different age and gender group

Age (year)	Number, n	DM			HT			DM with HT complication		
		Male	Female	Total	Male	Female	Total	Male	Female	Total
18–25	495	3.9	4.5	4.2	10.5	9.7	10.1	2.2	3.4	2.8
26–30	557	1.6	3.0	2.3	8.2	4.0	5.9	1.2	0.7	0.9
31–35	640	2.2	3.6	3.0	8.7	6.3	7.3	1.4	3.0	2.3
36–40	861	1.2	3.2	2.4	6.3	9.1	8.0	0.3	1.5	1.0
41–45	1437	3.3	3.1	3.2	9.2	9.6	9.5	0.7	0.9	0.8
46–50	1820	3.9	4.6	4.3	13.0	17.3	15.7	1.5	2.3	2.0
51–55	1355	6.7	7.2	7.0	15.5	26.0	22.3	2.5	3.6	3.2
56–60	1689	8.5	9.9	9.3	21.1	27.7	25.0	4.4	4.7	4.6
61–65	1741	6.7	12.9	10.1	31.3	33.3	32.4	3.7	7.1	5.6
66–70	1311	8.8	12.6	11.1	31.1	37.4	34.8	4.8	7.6	6.4
71–75	904	8.3	12.9	11.0	41.2	42.9	42.1	6.2	9.3	8.0
76–80	619	8.0	10.8	9.5	37.8	41.1	39.6	4.2	7.2	5.8
> 80	360	9.7	8.7	9.2	33.9	43.1	38.9	5.5	8.2	6.9
Total	13,789	5.8	7.8	7.0	21.0	23.9	22.7	3.0	4.4	3.8

The association of demographic factors and obesity with DM and HT was shown in Table 4. In general, older age, women, education with college or above, unmarried status, obesity, and central obesity were risk factors of all three diseases (*P* < 0.05). In addition, education in middle school was associated with increased risks of DM with HT complication (*P* = 0.013), and divorced or widowed status was associated with decreased risks of DM (*P* = 0.006).

**Table 4:** Associations of demographic factors and obesity with DM and HT

Variables	DM		HT		DM with HT complication	
	OR (95% CI)	*P*	OR (95% CI)	*P*	OR (95% CI)	*P*
**Age**	1.04 (1.03, 1.05)	< 0.001	1.06 (1.05, 1.06)		1.05 (1.04, 1.06)	< 0.001
**Gender**						
Men	Ref.		Ref.		Ref.	
Women	1.35 (1.17, 1.56)	< 0.001	1.16 (1.07, 1.27)	0.001	1.47 (1.22,1.79)	< 0.001
**Educational level**						
Primary school or below	Ref.		Ref.		Ref.	
Middle school	1.11 (0.96, 1.29)	0.153	0.10 (0.91, 1.09)	0.931	1.28 (1.05, 1.55)	0.013
College or above	1.38 (1.06, 1.79)	0.016	1.48 (1.25, 1.75)	< 0.001	2.10 (1.52, 2.90)	< 0.001
**Marital status**						
Married	Ref.		Ref.		Ref.	
Unmarried	1.67 (1.16, 2.40)	0.006	1.56 (1.23, 1.98)	< 0.001	2.53 (1.62, 3.95)	< 0.001
Divorced or widowed	0.76 (0.62, 0.92)	0.006	0.91 (0.80, 1.03)	0.133	0.83 (0.65, 1.06)	0.139
**Obesity**						
Yes	1.34 (1.10, 1.65)	0.005	1.57 (1.37, 1.81)	< 0.001	1.53 (1.19,1.97)	0.001
No	Ref.		Ref.		Ref.	
**Central obesity**						
Yes	2.05 (1.78, 2.37)	< 0.001	2.13 (1.94, 2.35)	< 0.001	2.57 (2.12, 3.10)	< 0.001
No	Ref.		Ref.		Ref.	

### Prevalence of CVDs by DM and HT

Table 5 showed the prevalence of CVDs by DM and HT. The overall self-reported prevalence was 1.4% for stroke, 7.5% for CHD, and 8.3% for total CVDs. The prevalence of total CVDs was 3.7% for non-DM & non-HT group, 12.1% for DM & non-HT group, 21.2% for non-DM & HT group, and 31.4% for DM & HT group. The difference in prevalence among the four groups was highly significant (*P* < 0.001). A similar difference was observed for stroke and CHD (*P* < 0.001).

**Table 5:** Prevalence of CVDs by the status of DM and HT (*n* = 14,422)

Prevalence (%)	Total	-DM & -HT(n_0_ = 10,709)	+DM & -HT(n_1_ = 548)	-DM & + HT(n_2_ = 2710)	+DM & + HT (n_3_ = 455)	*χ*^2^	P
stroke	**1.4 (209)**	0.6 (61)	2.4 (11)	3.9 (107)	5.5 (30)	241.685	< 0.001
CHD	**7.5 (1077)**	3.4 (361)	10.5 (48)	18.8 (510)	28.8 (358)	1133.675	< 0.001
Total CVD	**8.3 (1201)**	3.7 (399)	12.1 (55)	21.2 (575)	31.4 (172)	134.737	< 0.001

^**#**^ () Frequency in brackets

### Association of DM and HT with CVDs

Table 6 showed the associations of CVDs with DM and HT after adjusting for potential confounding factors (gender, age, education level, marital status, smoking, drinking, regular exercise, obesity, and central obesity for CHD and total CVDs; age, marital status, smoking, drinking, regular exercise, and central obesity for stroke). Comparing with the non-DM & non-HT group, an significantly increased risk of stroke, CHD, and total CVDs was observed among the DM & non-HT group, the non-DM & HT group, and the DM & HT group.

**Table 6:** Association of CVDs with DM & HT^#^

CVD status	OR	95% CI	Wald ***χ***^***2***^_**multiplicative**_	***P***_**multiplicative**_
**Total CVD** ^***Model 1***^				
-DM & -HT	Ref.	Ref.		
+ DM & -HT	2.53	(1.81, 3.55)		
-DM & + HT	4.35	(3.72, 5.09)		
+ DM & + HT	7.51	(5.86, 9.63)		
DM × HT			3.321	0.068
**Stroke** ^***Model 2***^				
-DM & -HT	Ref.	Ref.		
+ DM & -HT	2.71	(1.20, 6.14)		
-DM & + HT	4.78	(3.20, 7.14)		
+ DM & + HT	5.25	(2.93, 9.40)		
DM × HT			3.368	0.066
**CHD** ^***Model 3***^				
-DM & -HT	Ref.	Ref.		
+ DM & -HT	2.44	(1.72, 3.45)		
-DM & + HT	4.12	(3.50, 4.84)		
+ DM & + HT	7.49	(5.82, 9.64)		
DM × HT			1.815	0.178

^**#**^ Gender, age, education level, marital status, smoking, drinking, regular exercise, obesity, and central obesity were adjusted for in ***Model 1*** and ***Model 3***; Age, marital status, smoking, drinking, regular exercise, and central obesity were adjusted for in ***Model 2***; *Ref*, reference.

### Interaction effects of DM and HT on CVDs

We added the interaction term (DM × HT) into logistic models and found that the multiplicative interaction of DM and HT on CVDs was not found *(*Table 6*)*. We further evaluated the additive interaction of DM and HT *(*Table 7*)* and found the additive interaction was statistically significant for CHD (SI = 1.43, 95 CI, 1.03–1.97; RERI = 1.94; 95% CI, 0.05–3.83; AP = 0.26; 95% CI, 0.06–0.46) while the additive interaction on stroke was not significant.

**Table 7:** Additive interaction of DM and HT on cardio-cerebrovascular diseases

CVDs status	RERI		AP		SI	
	estimate	95% CI	estimate	95% CI	estimate	95% CI
Total CVDs	1.63	(−0.25, 3.51)	0.22	(0.01, 0.43)	1.33	(0.97, 1.83)
CHD	1.94	(0.05, 3.83)	0.26	(0.06, 0.46)	1.43	(1.03, 1.97)
stroke	−1.25	(−4.71, 2.22)	−0.24	(−0.97, 0.50)	0.77	(0.38, 1.60)

## Discussion

### Main finding

The prevalence was 22.7% for hypertension, 7.0% for diabetes, both were similar to the National population survey [17, 18]. Meanwhile, the prevalence of coronary heart disease, stroke, and total cardio-cerebrovascular diseases was 7.5, 1.4, and 8.3%, respectively. They were all lower than the previously reported prevalence especially stroke [19]. Older age, women, higher educational level, unmarried status, and obesity (central obesity) were risk factors of diabetes and hypertension. Participants with both diabetes and hypertension had a significantly increased risk of cardio-cerebrovascular diseases as compared with participants with only one condition. A significant synergistic additive interaction of diabetes and hypertension on coronary heart disease was observed.

### Comparisons with previous studies

Prior studies [20–22] have found that the comorbidity of hypertension and diabetes increased the risk of cardiovascular diseases dramatically, but their interaction was not reported [23–25]. Yun Ju Lai [26] found a synergism of diabetes and hypertension only among elderly women aged over 65 years old. In another cross-sectional study with a small sample size (886 participants), Cai [27] and colleagues found an interaction on the severity of stroke. However, all of those studies only evaluated multiplicative interaction.

As pointed out by Rothan [28], the additive interaction model is closer to the nature of biological interaction and has more relevant public health significance than the multiplication model. Vandenbroucke et al [29] suggested that both additive and multiplicative interaction should be reported when evaluating interactions. Even when two factors have no multiplicative interaction, they may have a positive interaction in the additive model [30]. This study reported both results of multiplicative and additive models. In the multiplicative model, the interaction items were not statistically significant but in the additive model, the interaction effects were positive.

### Potential explanations

It was reported that DM and HT shared common risk factors and pathophysiological pathways which were interconnected into a network and may even lead to a vicious cycle. Therefore, HT and DM are the main parts of the metabolic process of metabolic syndrome and they are prone to comorbidity [31]. Cardio-cerebrovascular diseases are multifactorial diseases. The risk of occurrence depends not only on the severity of a certain determinant but also on the number of determinants possessed by the individual [32]. Jonathan N [33] found that the combination of DM and HT has adverse effects on left ventricular structure, myocardial dysfunction, and arterial stiffness. Cesare Russo [34] found that HT and DM are independently associated with impaired left ventricular diastolic function. Their coexistence resulted in the most severe effect on left ventricular diastolic mechanics and was associated with higher left ventricular filling pressures than patients with one condition alone. Both DM and HT are crime culprits for atherosclerosis and are essential parts of the formation and aggravation of endothelial and smooth muscle function [35]. The combination of DM and HT can promote endothelial cell dysfunction [36]. The dysfunction of endothelial cells may change in the early stage of atherosclerosis. Both DM and HT can promote the generation of oxygen-derived free radicals, thus damaging endothelial function. When the two coexist, endothelial cell function further decreases, and smooth muscle function is also impaired [35]. Besides, the combination of DM and HT can promote monocyte adhesion to endothelial cells, thus increasing the production of vascular superoxide and the expression of monocyte chemoattractant protein-1 [37], leading to atherosclerosis and subsequent cardio-cerebrovascular diseases. In conclusion, recent studies show that there is a great biological possibility of interaction between diabetes and hypertension. However, the specific mechanism and degree of interaction remain unclear, thus further study is still warranted.

### Strengths and limitations

This is the first community-based cross-sectional study with a large sample size (14,422 participants) that investigated the interaction of diabetes and hypertension on cardio-cerebrovascular diseases. Both multiplicative and additive interactions were evaluated, and the results were consistent in theory, which provided strong support for the main conclusion. The multivariable logistical regression models in this study were adjusted for potential confounding factors according to the variable selection principle of DAG, which greatly improved the reliability of the results. This study adopted a cross-sectional design, which precluded causal correlations, and the information about the disease was provided by the investigators themselves, thus recall bias cannot be avoided. The prevalence of stroke, CHD, and CVD are low so that the interaction effects could be underestimated. In particular, this may be the reason for the tendency to null of the interaction on stroke to some extent, because the prevalence of stroke observed is only 1.4%. More prospective cohort studies will be needed in the future to prove this correlation and adjusted more confounders such as disease types, degree, treatment, and control status.

## Conclusions

DM combined with HT significantly increased the risk of cardio-cerebrovascular diseases and had a significant synergistic interaction effect on coronary heart disease. Participants who were old, women, highly educated, unmarried, and obese (central obese) had a high risk of diabetes and hypertension that we should take interventions to prevent the occurrence of cardio-cerebrovascular diseases. Also, since this study is a cross-sectional study at a single time point, causality cannot be confirmed. Therefore, more prospective cohort studies should be carried out in the future to confirm this conclusion.

## Supplementary Information

**Additional file 1: Appendix 1**. Questionnaire -- extract

## Data Availability

Not publically available except for reasonable requests by contacting the corresponding author.

## Abbreviations

HT: Hypertension
DM: Diabetes
CHD: Coronary heart disease
CVDs: Cardio-cerebrovascular diseases
