# Supplementary material for: Prevalence of diabetes and hypertension and their interaction effects on cardio-cerebrovascular diseases: a cross-sectional study
Source: BMC Public Health. 2021 Jun 25;21:1224. doi: 10.1186/s12889-021-11122-y (PMC8229421; doi:10.1186/s12889-021-11122-y)
Supplement: Supplementary file 1 — Additional file 1: Appendix 1. Questionnaire -- extract [file 12889_2021_11122_MOESM1_ESM.docx]

*Appendix 1*

**Questionnaire -- extract**

We intercepted the main questionnaire information used in this study, the specific content is as follows:

**Dear participants：**

We are investigators of Changsha center for Disease Control and prevention. To acquire the prevalence of chronic diseases among residents in our city, we are now collecting the following information. You are informed to fill this questionaire as required. If you have any questions in the process of filling in, you can consult the investigator. We promise that the information collected in this survey is only for writing investigation report and scientific research, and will not disclose your privacy or participate in any commercial activities. If you have any inconvenience, you have the right to withdraw from the investigation at any time. Thanks for your support and cooperation！

**Basic information**

1.What's your gender?

A. Male B. female

2. Your age? I'm years old.

3. Your marital status?

A. Unmarried B. married C. divorced

4. What is your education level?

A. Primary school and below B. junior high school C. senior high school / technical secondary school / junior college D. university or above

5. What's your occupation?

A. Agricultural, forestry, animal husbandry, fishery, and water conservancy production personnel

B. Person in charge of state organs, Party mass organizations, enterprises, and institutions

C. Professional and technical personnel

D. Clerks and related personnel

E. Business and service personnel

F. Production and transportation equipment operators and related personnel

G. Unemployed or unemployed

H. Retirement

I. Other occupations

**Disease information (Please answer the following questions according to the clear diagnosis of doctors in the secondary level above hospitals)**

6. Have you ever been diagnosed with diabetes? (including type 1 diabetes and type 2 diabetes).

A. Yes

B. No

7. Have you ever been diagnosed with hypertension?

A. Yes

B. No

8. Have you ever been diagnosed with coronary heart disease? (including ischemic coronary heart disease, myocardial infarction, angina pectoris, coronary artery bypass grafting, percutaneous coronary thrombolysis, coronary angioplasty).

A. Yes

B. No

9. Have you ever been diagnosed with stroke? (including ischemic stroke, hemorrhagic stroke, and transient ischemic attack).

A. Yes

B. No

**Living habits**

1. Do you smoke now?
2. Smoking (at least one cigarette per day)
3. former smoking (quit smoking more than 12 months)

C. never smoking

11. Do you drink now?

A. Yes (drink once or more in the recent month).

B. No

12. Do you exercise regularly?

A.Yes (exercise at least three times a week)

B.No

Thank you again for your cooperation！
